# Effective MR Molecular Imaging of Triple Negative Breast Cancer With an EDB-Fibronectin-Specific Contrast Agent at Reduced Doses

**DOI:** 10.3389/fonc.2019.01351

**Published:** 2019-12-03

**Authors:** Nadia R. Ayat, Amita Vaidya, Grace A. Yeung, Megan N. Buford, Ryan C. Hall, Peter L. Qiao, Xin Yu, Zheng-Rong Lu

**Affiliations:** ^1^Department of Biomedical Engineering, School of Engineering, Case Western Reserve University, Cleveland, OH, United States; ^2^Case Comprehensive Cancer Center, Case Western Reserve University, Cleveland, OH, United States

**Keywords:** EDB-fibronectin, GBCAs, targeted MRI contrast agent, breast cancer, tumor microenvironment

## Abstract

MR molecular imaging (MRMI) of abundant oncogenic biomarkers in tumor microenvironment has the potential to provide precision cancer imaging in high resolution. Extradomain-B fibronectin (EDB-FN) is an oncogenic extracellular matrix protein, highly expressed in aggressive triple negative breast cancer. A targeted macrocyclic gadolinium-based contrast agent (GBCA) ZD2-N_3_-Gd(HP-DO3A) (MT218), specific to EDB-FN, was developed for MRMI of aggressive breast cancer. The effectiveness of different doses of MT218 for MRMI was tested in MDA-MB-231 and Hs578T human triple negative breast cancer models. At clinical dose of 0.1 and subclinical dose of 0.04 mmol Gd/kg, MT218 rapidly bound to the extracellular matrix EDB-FN and produced robust tumor contrast enhancement in both the tumor models, as early as 1–30 min post-injection. Substantial tumor enhancement was also observed in both the models with MT218 at doses as low as 0.02 mmol Gd/kg, which was significantly better than the clinical agent Gd(HP-DO3A) at 0.1 mmol Gd/kg. Little non-specific enhancement was observed in the normal tissues including liver, spleen, and brain for MT218 at all the tested doses, with renal clearance at 30 min. These results demonstrate that MRMI with reduced doses of MT218 is safe and effective for sensitive and specific imaging of aggressive breast cancers.

## Introduction

MRI is a powerful non-invasive imaging modality that provides high resolution, three-dimensional images of soft tissues, including cancerous lesions. Gadolinium (III)-based contrast agents (GBCAs) are routinely used to enhance the contrast between cancerous lesions and surrounding normal tissues for accurate cancer detection and diagnosis (1). However, the use of clinical GBCAs for diagnosis and therapeutic monitoring is limited due to the lack of disease-specific contrast agents, and concerns of potential Gd toxicity (2–4). Currently used clinical GBCAs are non-specific to tumor tissues and are unable to accurately detect and differentiate aggressive malignancies from benign lesions. Therefore, high dosages of GBCAs are often used for detectable contrast enhancement in tumors. Frequent use of GBCAs at high doses may lead to adverse side effects such as nephrogenic systemic fibrosis (NSF) caused by the retention of Gd in tissues in patients with compromised renal function (2). In addition, GBCAs have been shown to deposit in human brains with intact blood brain barriers and in the absence of intracranial abnormalities (5). While no adverse pharmacological and pathological effects have been associated with the observed tissue deposition, it is clinically imperative to address this concern by developing tumor-specific targeted contrast agents that can be used at substantially reduced dosages (1). This would improve diagnostic imaging of cancer with MRI, and simultaneously minimize potential dose-dependent toxicity and non-specific organ accumulation to alleviate the safety concerns for clinical use of GBCAs.

While MRI is routinely used to obtain specific and high-resolution images of cancer tissues, developing clinically feasible targeted MRI contrast agents for MR molecular imaging (MRMI) of the biomarkers expressed on cancer cell surface is challenging, due to the low sensitivity of MRI and low concentration of the biomarkers. To address these challenges for clinical MRMI, we have explored a strategy to target abundant oncoproteins expressed in tumor extracellular matrix (ECM) for safe and effective MRMI of aggressive cancers (6, 7). Fibronectin (FN) is considered as a marker of epithelial-to-mesenchymal transition (EMT), which is a biological process responsible for invasion and metastasis of aggressive tumors, including breast cancer (8). We previously developed small molecular peptide-targeted MRI contrast agents specific to fibrin-fibronectin complexes for cancer MRMI. The targeted contrast agents produced robust contrast enhancement in aggressive tumors for effective cancer MRMI, and have shown the potential to detect micrometastases as small as 300 μm in size (9–11). Clinical evidence has demonstrated that the tumor-specific isoform of FN, extradomain-B fibronectin (EDB-FN) is specifically overexpressed in the ECM of aggressive tumors, including breast tumors, thereby making EDB-FN a promising target for early detection and differential diagnosis of breast cancer (12–14). Recently, we have designed a new generation of small peptide targeted MRI contrast agents to target EDB-FN, for precision MRMI of prostate and breast tumors (15–17). A small peptide specific to EDB-FN, named ZD2 (Thr-Val-Arg-Thr-Ser-Ala-As), was identified and used for the design of a targeted MRI contrast agent for MRMI of EDB-FN in cancer (8, 15, 18). MRMI of EDB-FN with the targeted contrast agents was effective for detection and risk-stratification of aggressive solid tumors, including breast and prostate cancer, in animal tumor models (15, 17).

We further optimized the ZD2 peptide targeted MRI contrast agent and developed ZD2-N3-Gd(HP-DO3A) (MT218) as a lead agent for cost-effective clinical translation (19). MT218 possessed higher T_1_ relaxivity (5.44 mM^−1^ s^−1^) than the previously reported ZD2-Gd(HP-DO3A) (*r*_1_ = 4.12 mM^−1^ s^−1^) (15, 19). At the clinical dose of 0.1 mmol Gd/kg, MT218 generated robust signal enhancement in aggressive solid tumors, allowing for effective MRMI for early detection and risk-stratification of prostate cancer. In this study, we investigated the dose-dependent effectiveness of MT218 for MRMI of two independent triple negative breast cancer (TNBC) xenografts in athymic mice. The expression of EDB-FN was assessed in triple negative MDA-MB-231 and Hs578T breast cancer cells and tumors. Specific tumor contrast enhancement with MRMI was tested using clinical and subclinical doses of MT218 (as low as 0.02 mmol Gd/kg) in these tumor models, in comparison with a clinical agent Gd(HP-DO3A). A minimally effective dose was identified for effective MRMI of TNBC and for minimizing potential dose-dependent side-effects associated with GBCAs. Non-specific tissue enhancement was also evaluated in normal organs and tissues in the animal models.

## Materials and Methods

### Cell Lines and Reagents

MDA-MB-231 and Hs578T TNBC cells were purchased from ATCC (Manassas, VA). MDA-MB-231 cells were maintained in Dulbecco's Modified Eagle's Medium (DMEM, Gibco, Waltham, MA) supplemented with 10% fetal bovine serum (FBS, Sigma, St. Louis, MO) and 1% Penicillin/Streptomycin. Hs578T cells were cultured in DMEM supplemented with 10% FBS, 1% Penicillin/Streptomycin, and 0.01 mg/mL recombinant human insulin (Sigma). MCF7 cells were maintained in Eagle's Minimum Essential Medium (EMEM, Gibco) supplemented with 10% FBS (Sigma), 1% Penicillin/Streptomycin, and 0.01 mg/mL recombinant human insulin (Sigma). MDA-MB-231 and Hs578T cells were engineered to stably express firefly luciferase and GFP with a lentivirus encoding for CMV-Luciferase (Firefly)-2A-GFP (Neo) (Amsbio, Cambridge, MA) and selected using flow cytometry.

### Semiquantitative Real-Time PCR Analyses

Quantitative real-time PCR was conducted as described previously (20). Briefly, total RNA was extracted from TNBC cells using an RNeasy Plus Mini Kit (Qiagen, Germantown, MD) as per manufacturer's instructions. Reverse transcription was performed using the miScript II RT Kit (Qiagen), followed by qPCR using miScript SYBR Green PCR kit (Qiagen). mRNA expression levels were normalized to 18S control. The following primers were purchased from Integrated DNA Technologies (Coralville, IA): 18S: Fwd 5′-TCAAGAAC GAAAGTCGGAGG-3′ and Rev 5′-GGACATCTAAGGGCATCACA-3′; EDB-FN: Fwd 5′- AGCCCTGTGACTGTGTAGTA-3′ and Rev 5′-AGCCCTGTGACTGTGTAGTA-3′.

### *In vitro* EDB-FN Binding

An EDB-FN-specific fluorescent probe ZD2-Cy5.5 was synthesized as previously described (8). Approximately 300,000 breast cancer cells were plated onto iBidi glass bottom plates coated with Corning Matrigel Membrane Matrix (Corning, Corning, NY). Upon formation of tumor spheroids, ZD2-Cy5.5 (250 nM) along with 5 μg/mL Hoechst 33342 (Invitrogen, Carlsbad, California) were incubated with the spheroids for 1 h. Peptide binding was monitored using an Olympus FV1000 confocal microscope (Olympus Corporation, Tokyo, Japan).

### Animal Models

Female nude mice (nu/nu Balb/c background, 4–6 weeks old) were purchased from Jackson Laboratories (Bar Harbor, Maine) and cared for in the Animal Core Facility at Case Western Reserve University (Cleveland, OH). All animal experiments were conducted in accordance with an approved protocol by the IACUC of CWRU. Mammary fat pads were injected with 2 x 10^6^ MDA-MB-231-GFP-Luc and 4 × 10^6^ Hs578T-GFP-Luc cells suspended in a matrigel-PBS mixture (1:1). Tumor volumes were monitored weekly using a Vernier caliper. Once the average tumor volume reached 70–90 mm^3^, mice were subjected to MR imaging (*n* = 5 for MT218 and *n* = 5 for Gd(HP-DO3A) (Bracco, Milan, Italy).

### MR Molecular Imaging

MR molecular imaging was performed on a 3T MRS 3000 scanner (MR Solutions, Surrey, UK). T_1_-weighted images were acquired pre- and post-injection of MT218 at doses of 0.1 mmol Gd/kg (clinical dose), 0.04 mmol Gd/kg and 0.02 mmol Gd/kg using a fast spin echo (FSE) axial sequence with respiratory gating (T_R_ = 305 ms, T_E_ = 11 ms, FOV = 40 × 40 mm, slice thickness = 1 mm, slice number = 15, N_av_ = 2, matrix = 256 × 256) using a mouse short quad coil (MR Solutions). Biodistribution studies were conducted using a FSE coronal sequence with respiratory gating (T_R_ = 305 ms, T_E_ = 11 ms, FOV = 90 × 90 mm, slice thickness = 1 mm, slice number = 20, N_av_ = 2, matrix = 248 × 512). A group of 5 tumor-bearing mice was imaged for each dose. Images were acquired before and at 10, 20, and 30 min post-injection. The imaging protocol was repeated with the clinical agent, Gd(HP-DO3A) as a control at 0.1 mmol Gd/kg.

The MRI data were exported into DICOM data and analyzed using FIJI (https://imagej.net/Fiji). The contrast-to-noise ratio (CNR) of tumors were calculated at 10, 20, and 30 min post-injection as the difference of the mean tumor intensity and the mean muscle intensity, divided by the standard deviation of the noise. Tumor signal to noise ratio (SNR) was obtained as the mean signal intensity of the tumor divided by the standard deviation of the noise at 20 min post-contrast injection.

### Histological Analysis

Tissue fixation, sectioning, and H&E staining were performed at the tissue resource core at Case Western Reserve University (Cleveland, OH). Tumor sections were deparaffinized and rehydrated (Abcam, Cambridge, UK) and blocked in PBS-T containing goat serum (10%) for 1 h. Rabbit anti-EDB-FN antibody, G4, (Absolute Antibody, Boston, MA) diluted with PBS-T containing goat serum (1:500) was applied to the tissue and incubated at room temperature for 1 h. After 3 washes of 5 min each, AlexaFluor555-conjugated anti-rabbit IgG secondary antibody (1:2,000, Abcam) was incubated with tissue sections for 1 h. Tissue sections were counterstained with AbcamProlong Gold with DAPI (Abcam). Fluorescence images were obtained using an Olympus FV1000 confocal microscope. H&E images were acquired using an Olympus Bx61VS slide scanner (Olympus America, Center Valley, PA) and processed in OlyVIA software.

### Immunoblotting

MDA-MB-231 and Hs578T tumors were collected and homogenized in 350 μL 1x RIPA buffer supplemented with protease inhibitor (Roche, Basel, Switzerland). Samples were subsequently mixed with 350 μL 1x Laemmli buffer solution and boiled for 10 min, vortexing every 5 min. Samples were centrifuged at 15,000 g for 15 min at 4°C to remove insoluble components. Protein concentrations were quantified using Lowry assay with bovine serum albumin as a standard. Protein extracts (40 μg) were loaded onto a 4–20% gradient gel (BioRad, Hercules, CA) for SDS-PAGE, and transferred onto a nitrocellulose membrane (Cell Signaling, Danvers, MA). The nitrocellulose membrane was incubated overnight with anti-EDB-FN antibody G4 (1:1,000) and loading control anti-β-actin (Cell Signaling; 1:1,000). Gels were visualized using a ChemiDoc XRS system (BioRad).

### Statistical Analysis

All experiments were independently replicated at least three times unless otherwise stated. Statistical comparison of the different dosing groups was performed using Graphpad software. Statistical significance between two groups was calculated using an unpaired *t*-test. Data between three groups was compared using one-way ANOVA with *p* < 0.05 being statistically significant.

## Results

**Figure 1:**
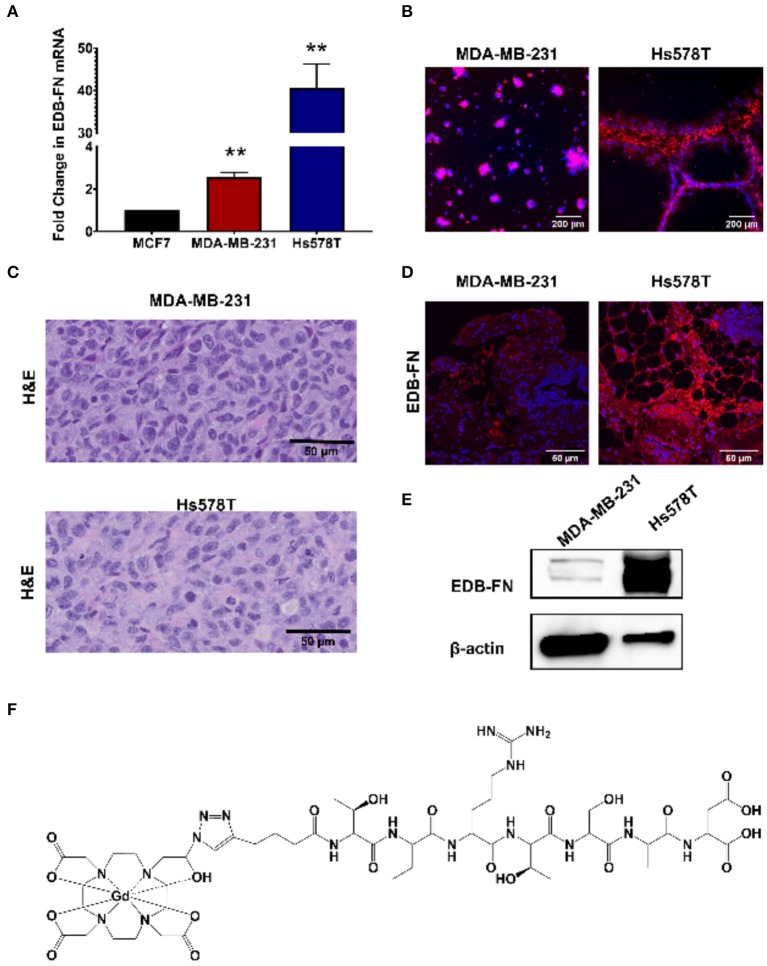
EDB-FN is overexpressed in poorly differentiated, triple negative breast cancer. **(A)** Endogenous EDB-FN mRNA levels is significantly elevated in triple negative breast cancer cell lines MDA-MB-231 and Hs578T (claudin-low ER^−^, PR^−^, HER2^−^) compared to MCF7 (luminal A; ER^+^, PR^+/−^, HER2^−^) [**p < 0.01, (22)]. mRNA was normalized to 18S mRNA control. **(B)** Representative fluorescence imaging reveals enhanced ZD2-Cy5.5 (red) binding in 3D cultures of MDA-MB-231 and Hs578T cells. **(C)** H&E staining of MDA-MB-231 and Hs578T tumor sections. Scale bar: 50 μm. **(D)** Analysis of EDB-FN expression in MDA-MB-231 and Hs578T tumor sections. Scale bar: 50 μm **(E)** Western blot analysis of EDB-FN expression in MDA-MB-231 and Hs578T tumors. β-actin was used as a loading control. **(F)** Chemical structure of ZD2-N3-Gd(HP-DO3A) (MT218).

The triple negative MDA-MB-231 and Hs578T are highly invasive, mesenchymal breast cancer cell lines (21). Two independent mouse orthotopic xenografts were developed by inoculating the cancer cell lines into the mammary fat pads for assessing the effectiveness of MT218 at different doses. The expression of EDB-FN in the cell lines was first compared to a hormone receptor-positive epithelial breast cancer cell line MCF-7 as a less invasive control (17). As shown in Figure 1A, both MDA-MB-231and Hs578T cells had significantly higher EDB-FN mRNA expression (3.5- and 45-fold) when compared to MCF7 cells, respectively. Since EDB-FN is secreted into the tumor ECM, the expression of EDB-FN was also evaluated in 3D tumor spheroids of MDA-MB-231 and Hs578T cells incubated with ZD2-Cy5.5, a fluorescent peptide probe specific to EDB-FN. Both the 3D spheroids demonstrated strong ZD2-Cy5.5 binding, evidenced by the intense red fluorescence, indicating elevated expression and secretion of EDB-FN by the cancer cells (Figure 1B). Moreover, the Hs578T spheroids showed stronger ZD2-Cy5.5 staining and thus higher EDB-FN expression than the MDA-MB-231 spheroids, in consistence with the qRT-PCR results. The differential EDB-FN expression in the two cancer cell lines was also corroborated in their orthotopic tumor xenografts. Figure 1C shows histological sections of poorly differentiated H&E stained tumor tissues. Immunofluorescence staining of MDA-MB-231 and Hs578T tumor sections was performed using an EDB-FN specific G4 antibody. Strong EDB-FN expression was observed in Hs578T tumors when compared to the MDA-MB-231 tumor sections (Figure 1D). Western blot analysis also revealed abundant expression of EDB-FN in Hs578T tumors when compared to MDA-MB-231 tumors (Figure 1E, Supplementary Datasheet 1). These results demonstrate elevated expression of EDB-FN in both the triple negative breast cancers, more so in the Hs578T model.

**Figure 2:**
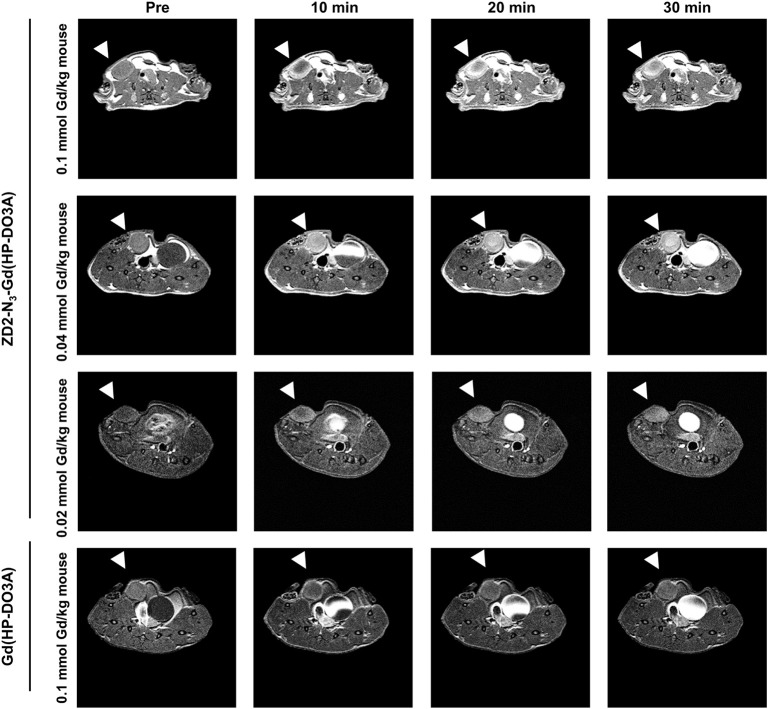
MRMI with MT218 of MDA-MB-231 tumors in mice. Representative axial T_1_-weighted 2D fast spin echo images taken before and at 10, 20, and 30 min post-injection of MT218 at doses of 0.1, 0.04, and 0.02 mmol Gd/kg mouse. Tumors are indicated by the white arrow heads. The clinical agent Gd(HP-DO3A) was used as a control at 0.1 mmol Gd/kg.

**Figure 3:**
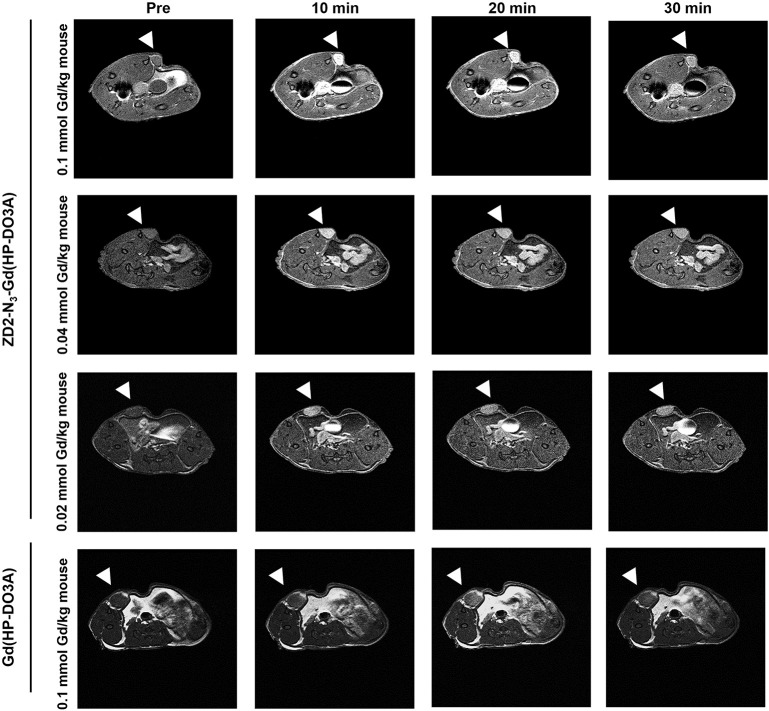
MRMI with MT218 of Hs578T tumors in mice. Representative axial T_1_-weighted 2D fast spin echo images taken before and at 10, 20, and 30 min post-injection of MT218 at doses of 0.1, 0.04, and 0.02 mmol Gd/kg mouse. Tumors are indicated by the white arrow heads. The clinical agent Gd(HP-DO3A) was used as a control at 0.1 mmol Gd/kg.

Following the characterization of EDB-FN expression, tumor contrast enhancement of MT218 was evaluated in mice bearing MDA-MB-231 and Hs578T xenografts before and after intravenous injection of MT218 at 0.1, 0.04, and 0.02 mmol Gd/kg. The chemical structure of MT218 is shown in Figure 1F. The tumor enhancement of the clinical control Gd(HP-DO3A) was also tested at the regular clinical dose of 0.1 mmol Gd/kg. Representative 2D T_1_-weighted spin-echo MR images of the tumors were obtained before and at different time-points after injection of the contrast agents. While the non-targeted clinical agent Gd(HP-DO3A) resulted in little signal enhancement (Figures 2, 3), MT218 exhibited dose-dependent tumor enhancement in both the tumors. Strong signal enhancement was observed in both MDA-MB-231 and Hs578T xenografts at clinical dose of 0.1 mmol Gd/kg of MT218. This signal brightness decreased slightly at a reduced dose of 0.04 mmol Gd/kg for the two tumors (Figures 2, 3). The enhancement was then reduced substantially at 0.02 mmol Gd/kg, but remained higher than that produced by clinical dose of Gd(HP-DO3A) (0.1 mmol Gd/kg) (Figure 2), demonstrating the superior tumor-targeting efficiency of MT218 over the clinical control. In addition, Hs578T tumors with higher EDB-FN expression exhibited brighter signal enhancement than MDA-MB-231 tumors at the same doses of MT218 (Figure 3). It appears that the background noise was low at the low doses for MT218. The tumor-specific contrast enhancement with MT218 in both the tumors lasted for at least 30 min for all the tested doses.

**Figure 4:**
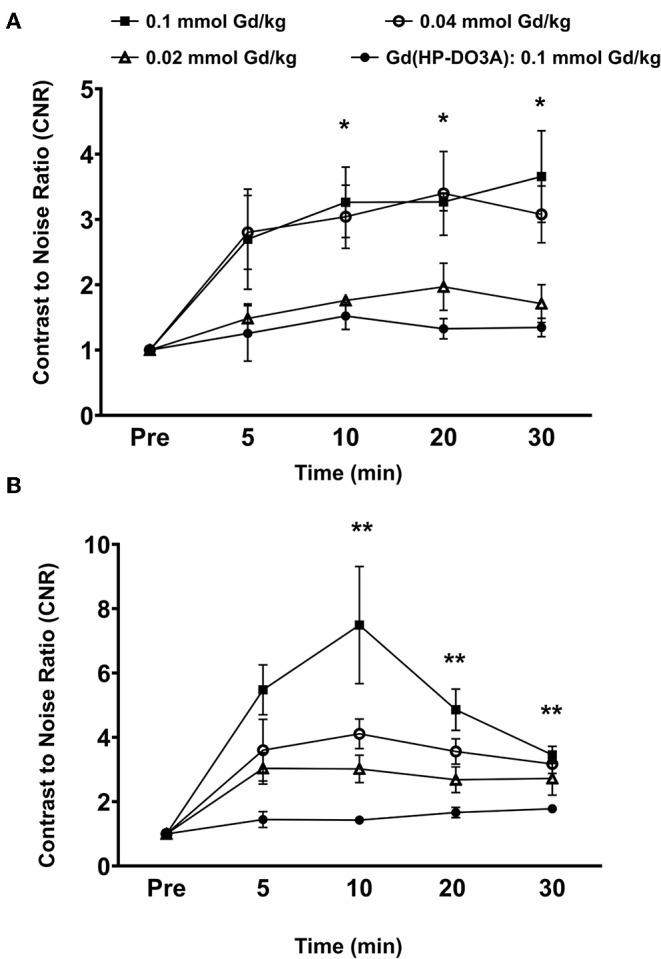
Analysis of fold increase of tumor contrast-to-noise ratio (CNR) from images acquired as indicated in MDA-MB-231 **(A)** and Hs578T **(B)** tumors. Data is represented as mean ± s.e.m. *n* = 5 for MDA-MB-231 and Hs578T tumors. *p < 0.05 for comparison of increased CNR ratio of 0.1 and 0.04 mmol Gd/kg to control Gd(HP-DO3A). No statistical significance was observed between 0.02 mmol Gd/kg MT218 to control. **p < 0.05 for comparison of increased CNR ratio of 0.1, 0.04, and 0.02 mmol Gd/kg to control.

Quantitative analysis of the tumor signal enhancement revealed different dose-dependent signal enhancement pattern of MT218 in the tumor models. Figure 4 shows the contrast-to-noise ratios (CNR) in the tumor models for up to 30 min post-injection of the agents. For the MDA-MB-231 model, MT218 produced comparable CNR at 0.1 and 0.04 mmol Gd/kg doses (Figure 4A), with ~3 to 3.5-fold CNR increase over their respective pre-contrast images and those with Gd(HP-DO3A). At 0.02 mmol Gd/kg, MT218 produced, slightly higher (1.5-fold CNR) than that of clinical control agent. For the Hs578T tumor model, MT218 showed a dose-dependent trend of tumor CNR, although it was not statistically significant (Figure 4B). Specifically, MT218 produced up to 7-, 4-, and 3-fold CNR increase at the doses of 0.1, 0.04, and 0.02 mmol Gd/kg, respectively; while Gd(HP-DO3A) only produced 1.3-fold CNR increase in the tumor. The different dose-dependent enhancement pattern of MT218 in the tumor models could be attributed to their differential EDB-FN expression levels. MT218 might show saturated binding in MDA-MB-231 tumors at the high 0.1 mmol Gd/kg dose because of relatively low EDB-FN expression in the tumor. However, relatively consistent CNR was observed in the MDA-MB-231 tumors for at least 30 min. In contrast, the Hs578T tumors had higher EDB-FN expression and showed time-dependent tumor CNR changes at 0.1 mmol Gd/kg (Figure 4B). MT218 showed maximum signal enhancement at 0.1 mmol Gd/kg at 10 min post-injection and gradual reduction of tumor CNR over time. It produced relatively consistent CNR in the Hs578T tumors at the low doses for up to 30 min. MT218 produced substantial tumor CNR in the first minute post-injection in both the tumors, especially at the high doses.

**Figure 5:**
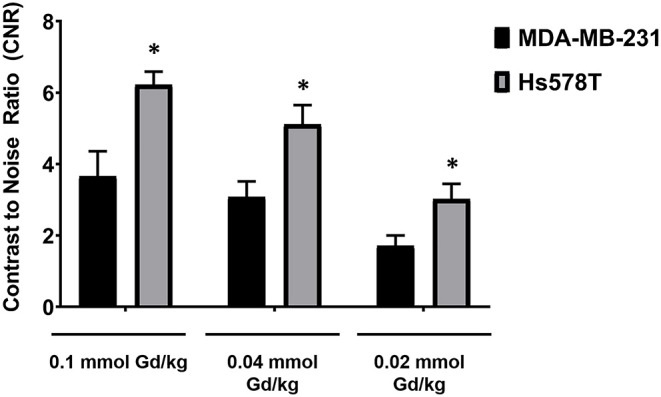
Analysis of tumor signal-to-noise ratio (SNR) at 20 min post-injection of MT218 at doses of 0.1, 0.04, and 0.02 mmol Gd/kg mouse. Data is represented as mean ± s.e.m. *n* = 5 for MDA-MB-231 and Hs578T tumors. *p < 0.05 for comparison of increased SNR ratio.

To determine if the uptake of MT218 is correlated with the endogenous EDB-FN expression in different tumor xenografts, we evaluated the changes in SNR in the MDA-MB-231 and Hs578T tumor models at 20 min post-injection of MT218 at 0.1, 0.04, and 0.02 mmol Gd/kg (Figure 5). At 0.1 mmol Gd/kg, the Hs578T tumors with high EDB-FN levels had 1.7-fold SNR over that of the MDA-MB-231 tumors with relatively lower EDB-FN (Figure 5). This tumor SNR decreased at the lower doses (0.04 and 0.02 mmol Gd/kg) in both the tumor models, with 1.7-fold higher SNR in Hs578T tumors than MDA-MB-231 tumors. On the other hand, no significant difference in SNR was observed for MT218 at different doses within the same tumor model. These results indicate that the uptake profile of MT218 is consistent with the tumor-specific expression profile of EDB-FN. Thus, MT218 has the promise to facilitate effective MRMI of aggressive tumors at doses as low as 0.02 mmol Gd/kg, a five-times reduction as compared to the clinical contrast agent.

**Figure 6:**
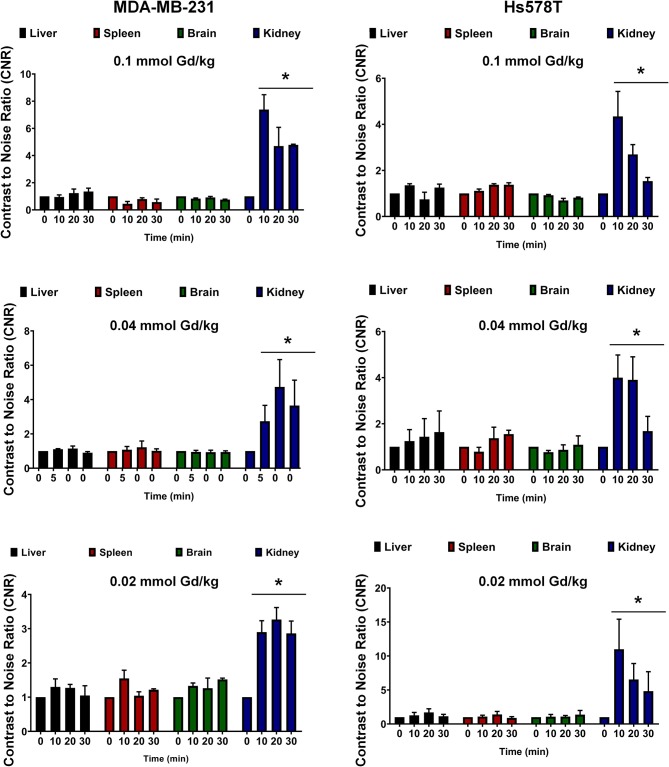
Contrast-to-noise ratio at 10, 20, and 30 min post-injection of MT218 at doses of 0.1, 0.04, and 0.02 mmol Gd/kg. Analysis of fold increase of CNR of liver, spleen, brain, and kidney were acquired in groups as indicated. Post-contrast CNRs of kidneys are significantly increased over pre-contrast (*p < 0.05).

In order to assess potential non-specific binding and enhancement of MT218, we analyzed the CNR in the brain and the key metabolic organs including the liver, kidney, and spleen. At all the tested doses of MT218, no significant changes in CNR were observed in the liver, spleen, or brain of all the tested mice for up to 30 min (Figure 6). Significant enhancement was observed in the kidneys as early as 10 min post-injection, but it steadily decreased at 30 min, indicating renal clearance of the agent. Taken together, these results exhibit the ability of MT218 to facilitate effective MRMI of aggressive tumors with minimal non-specific binding in normal tissues and organs at substantially reduced doses.

## Discussion

This work highlights the potential of the targeted contrast agent MT218 for effective MRMI of aggressive breast cancer at substantially reduced doses. MRMI is advantageous for precision cancer imaging if tumor-specific targeted contrast agents are available for risk-stratification of aggressive cancers (16, 23). MT218 targets the extracellular matrix oncoprotein EDB-FN, which is highly expressed in aggressive TNBC tumors. The ECM oncoprotein is more accessible for binding of the targeted contrast agent than cell surface biomarkers. Consequently, sufficient MT218 molecules bound to the abundant tumor-specific ECM protein produce robust enhancement throughout tumor tissues even at one fifth the clinical dose. Effective MRMI at such reduced doses has the promise to minimize the potential dose-dependent toxic side-effects of GBCAs in clinical practice, addressing the important safety concerns of their clinical use (2, 24).

MRMI with MT218 also exhibits several unique features for molecular imaging of TNBC tumors. MT218 produces significant tumor enhancement as early as 1 min post-injection at all the tested doses. High accessibility to EDB-FN in the tumor ECM allows rapid binding of MT218, resulting in effective tumor enhancement at the early time-point post-injection. MT218 produces relatively uniform contrast enhancement throughout the tumor tissues, especially at the later time-points post-injection. These observations indicate that EDB-FN is expressed throughout the ECM of the TNBC tumors, which enables specific binding of MT218 across the tumor tissues. The signal enhancement correlates well with the expression levels of EDB-FN in the tumors. EDB-FN is a marker of EMT and tumor angiogenesis (12). Clinical studies have shown that its expression is associated with poor survival of oral cancer patients (25, 26). Therefore, MRMI of EDB-FN with MT218 has the potential to differentiate tumor aggressiveness based on the EDB-FN expression (8, 15, 17).

GBCAs are FDA approved, and widely used for contrast-enhanced MRI for cancerous lesions (1). Although there have been observations of tissue accumulation and nephrogenic systemic fibrosis in patients with compromised renal functions for GBCAs, little clinical evidence exists to link their deposition to adverse effects. MRI contrast agents with other paramagnetic materials, such as iron and Mn, have been explored as alternative MRI contrast agents. Superparamagnetic iron oxide nanoparticles have been shown to provide excellent T_2_-weighted MR signal enhancement. Ferumoxytol, a carboxymethyldextran-coated iron oxide nanoparticle has been used as a contrast agent in lieu of GBCAs (27). However, ferumoxytol is large, and has a blood elimination half-life of 14 h, requiring patients to be imaged the day post-injection to observe tumor enhancement. Mn complexes have also been explored as GBCA alternatives due to their strong paramagnetic properties, (24, 28). However, frequent use of Mn-based contrast agents can also be associated with toxicity concerns, limiting their use clinically (29, 30). Consequently, comprehensive safety assessments are still required for these non-GBCAs before any approval for clinical use by the regulatory agents. The FDA has concluded that there is little evidence that directly links gadolinium retention to adverse side-effects in healthy patients, suggesting that the benefits of GBCAs outweigh any potential risks (31). Nevertheless, the ability to substantially reduce the dose of GBCAs through tumor-specific contrast agents will further enhance the safety profile of GBCAs for clinical use.

MT218 is a small peptide targeted macrocyclic contrast agent based on a clinical macrocyclic agent Gd(HP-DO3A), which has excellent thermodynamic and kinetic stability (19, 32, 33). Additionally, cyclic GBCAs have shown to have lower brain accumulation compared to linear contrast agents, indicating an excellent safety profile (34). It is expected that MT218 has a similar safety profile as the clinical agent. Comprehensive preclinical pharmacological and toxicity studies of MT218 are currently on-going based on FDA guidelines for clinical translation. To our knowledge, MT218 is the only targeted small molecular MRI contrast agent specific to an oncoprotein for effective MRMI of cancer. The clinical translation of MT218 will enable precision MRMI for the detection and risk-stratification of aggressive breast cancer. High resolution MRMI by MT218 could provide early detection of aggressive breast cancer and metastatic disease (14). Importantly, the administration of MT218 at reduced doses would alleviate the safety concerns associated with the dose-dependent side effects of GBCAs.

## Conclusions

EDB-FN is highly expressed by aggressive TNBC cells and tumors. The targeted MRI contrast agent MT218 specific to EDB-FN is effective for MRMI of TNBC tumors in mice at significantly reduced doses. MT218 rapidly binds to the extracellular matrix oncoprotein and produces robust tumor contrast enhancement at 1 min post-injection, lasting for 30 min. The effective MRMI with MT218 at low doses is an advantageous safety feature, and holds promise to minimize dose-dependent side effects of GBCAs. The clinical translation of MT218 has the potential to overcome the limitations of the existing clinical GBCAs for precision cancer MRMI and to provide accurate detection and risk-stratification of aggressive breast cancer.

## Data Availability Statement

The datasets generated for this study are available on request to the corresponding author.

## Ethics Statement

The animal study was reviewed and approved by CWRU IACUC.

## Author Contributions

The conceptual design of the research was designed by NA and Z-RL. NA participated in execution of all aspects of the research work. NA and XY designed all MR sequences used in this study. GY and MB participated in data analysis of MR images. AV, RH, and PQ participated in fluorescence imaging and acquisition. The manuscript was written and edited by NA and Z-RL. AV assisted with critical comments and edits for the manuscript.

### Conflict of Interest

Z-RL is a co-founder of Molecular Theranostics, LLC and has ownership interest in the company. The remaining authors declare that the research was conducted in the absence of any commercial or financial relationships that could be construed as a potential conflict of interest.
