# Supplementary material for: Effective MR Molecular Imaging of Triple Negative Breast Cancer With an EDB-Fibronectin-Specific Contrast Agent at Reduced Doses
Source: Front Oncol. 2019 Dec 3;9:1351. doi: 10.3389/fonc.2019.01351 (PMC6901824; doi:10.3389/fonc.2019.01351)
Supplement: Supplementary file 1 [file Data_Sheet_1.DOCX]

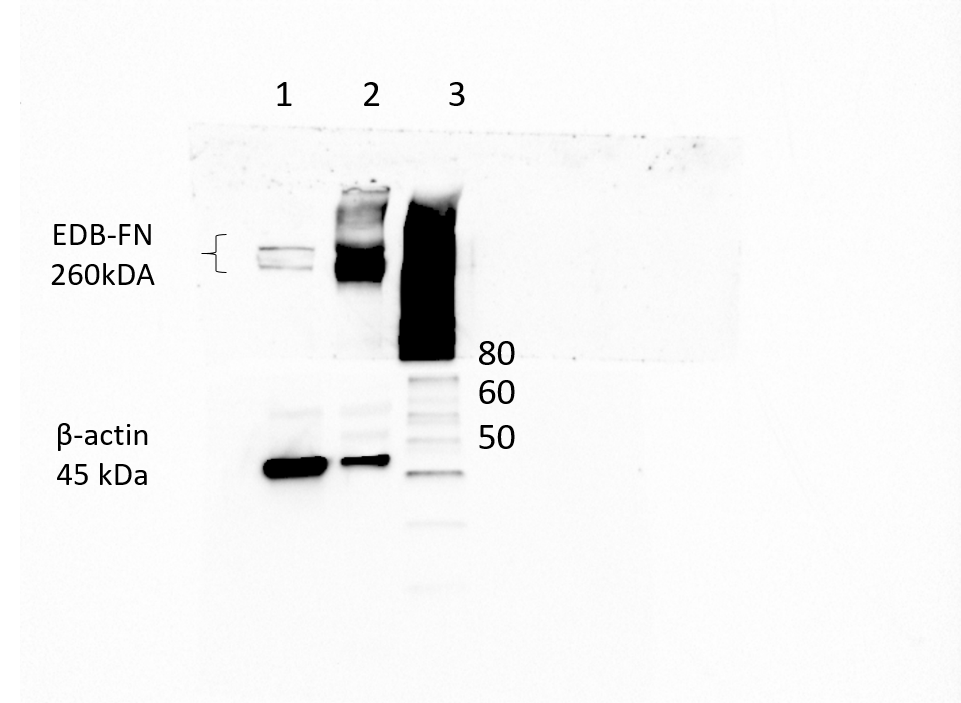


**Legend:** 1) MDA-MB-231 tumor 2) Hs578T tumor 3) MagicMarkXP

Exposure: 56.7seconds


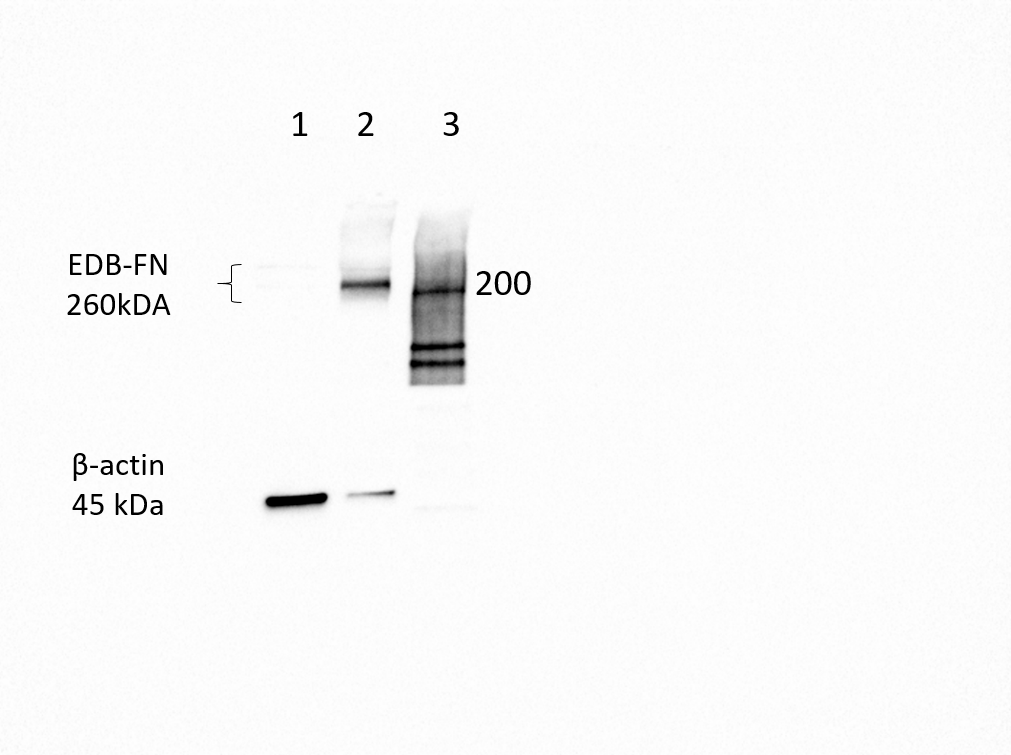


**Legend:** 1) MDA-MB-231 2) Hs578T 3) MagicMarkXP

Exposure: 1second
